# Investigating the Effect of Syringe Infiltration on *Nicotiana tabacum* (Tobacco)

**DOI:** 10.1021/acsagscitech.4c00170

**Published:** 2024-12-21

**Authors:** Cyril Routier, Carmen Hermida-Carrera, Eleni Stavrinidou

**Affiliations:** † Laboratory of Organic Electronics, Department of Science and Technology, Linköping University, SE-60174 Norrköping, Sweden; ‡ Umeå Plant Science Centre, Department of Forest Genetics and Plant Physiology, Swedish University of Agricultural Sciences, SE-90183 Umeå, Sweden

**Keywords:** plant infiltration technique, leaf physiology, thermography, stomatal conductance, photosynthesis

## Abstract

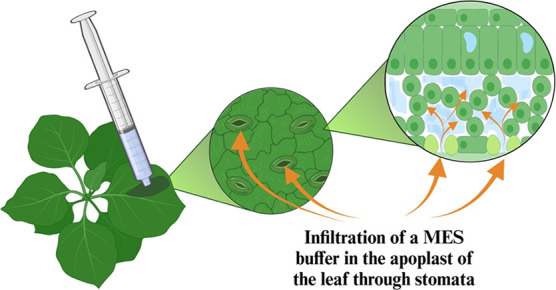

Plant infiltration techniques, particularly agroinfiltration, have transformed plant science and biotechnology by enabling transient gene expression for genetic engineering of plants or genomic studies. Recently, the use of infiltration has expanded to introduce nanomaterials and polymers in plants to enable nonnative functionalities. Despite its wide use, the impact of the infiltration process *per se* on plant physiology needs to be better understood. This study investigates the effect of syringe infiltration, a commonly employed technique in plants, using a typical infiltration buffer solution. Noninvasive and real-time monitoring methods, including high-resolution thermal imaging and a porometer/fluorometer, were used to study the physiological responses and stress levels of the infiltrated plants. Our results revealed localized cell damage at the infiltration site due to syringe compression, but the overall cell viability and tissue integrity were largely unaffected. Thermography showed a temporary temperature increase of the leaves and stomatal conductance alterations postinfiltration, with leaf recovery in 3–6 days. Additionally, fluorescence measurements indicated a 6% decrease in maximum quantum efficiency (*F*_v_/*F*_m_) and a 34% decrease in photosystem II (ΦPSII) quantum yield, persisting for 5 days after infiltration, suggesting sustained photosystem efficiency changes. Our work highlights the need to consider the effect of infiltration when performing biological studies and aims to facilitate the optimization of protocols commonly used in plant science and biotechnology.

## Introduction

Plant infiltration is a versatile technique commonly used in plant biology and biotechnology primarily for genetic engineering.^1^ Agroinfiltration specifically is the leaf infiltration with *Agrobacterium tumefaciens*, a bacterial pathogen that acts as a carrier of genetic material. This specific infiltration method has proven invaluable for a wide range of applications, including the introduction of foreign DNA allowing gene expression and analysis,^2−4^ pharmaceutical protein production^5,6^ and the study or enhancement of disease resistance in plants.^7,8^ Furthermore, the infiltration technique has recently emerged as a promising route for introducing functional materials such as nanoparticles or polymers into plant tissue, leveraging materials science and nanotechnology to enhance and augment plant functions.^9−11^ For example, quantum dots have been infiltrated into plants for precise and targeted delivery of chemicals to plants’ chloroplasts,^12^ while mesoporous silica nanoparticles have been used for potential delivery of various biomolecules.^13^ Functionalized nanotubes have also been infiltrated in plant tissue to enable in vivo detection of H_2_O_2_^14^ or to transform plants into environmental sensors for arsenic detection.^15^ Infiltration of conducting polymers has enabled the development of organic electronic devices integrated into plant tissue,^16^ while infiltration of polyethylenimine-based nanoparticles has been used to increase plants CO_2_ capture and storage.^17^

While there are several techniques for plant infiltration, one of the most commonly used is the local syringe infiltration, which involves directly injecting substances into the leaf apoplast using a needleless syringe.^18−20^ Despite its widespread use, there is a lack of understanding of the impact of the infiltration process *per se* on plant physiology. Most studies focus on how the infiltrated active substances and buffers affect the transient expression of genes in the plants or the plants targeted processes,^21,22^ while limited attention has been given to the effect of the infiltration on plant tissue and processes *per se*. However, a recent study focused on improving and standardizing the syringe infiltration process demonstrated that water or buffer infiltration induced a decrease in plant assimilation and photosynthetic efficiency for the days following the infiltration.^23^ Indeed, the introduction of a liquid in the leaf mesophyll might temporarily alter the water balance within the infiltrated region, affecting the humidity gradient around the leaf, which could potentially alter the rate of transpiration. Additionally, it could affect the osmotic balance within plant cells, impacting turgor pressure which can influence stomatal aperture, reducing the overall transpiration rate. While these effects are expected to be temporary, they highlight the need for careful consideration of the infiltration process and its potential consequences in plant studies.

In this study, we investigated the effect of syringe infiltration *per se* on *Nicotiana tabacum* (tobacco) transpiration and photosynthesis. We selected 2-(N-morpholino)ethanesulfonic acid (MES) as the infiltration medium due to its well-documented use in plant science and biotechnology.^24,25^ MES is a zwitterionic buffer that maintains stable pH in the range of 5.5 to 7.0, making it suitable for experiments requiring a slightly acidic and stable environment. Additionally, MES has low toxicity to plants and minimal interaction with metal ions, which ensures that it does not interfere with key physiological processes during experiments. Its chemical inertness allows for accurate study of plant transpiration and photosynthesis, while maintaining the integrity of plant tissues. MES has been successfully employed in various plant–related applications, including transient gene expression,^26−28^ virus-induced gene silencing,^29^ and nanoparticle and nanotube infiltration.^14,15,17^ Using thermal imaging, we monitored in real time the effect of infiltration on the leaves’ temperature over the course of several days and extracted the variations in relative stomatal conductance. Furthermore, we correlated the relative stomatal conductance with absolute values measured with a porometer, and we discuss deviations from direct proportionality. Finally, using a porometer/fluorometer, we evaluated the impact of infiltration on chlorophyll fluorescence parameters. Our findings offer insights into the effect of the infiltration process, which could inform future protocols and practices in plant biotechnology.

## Results and Discussion

**Figure 1:**
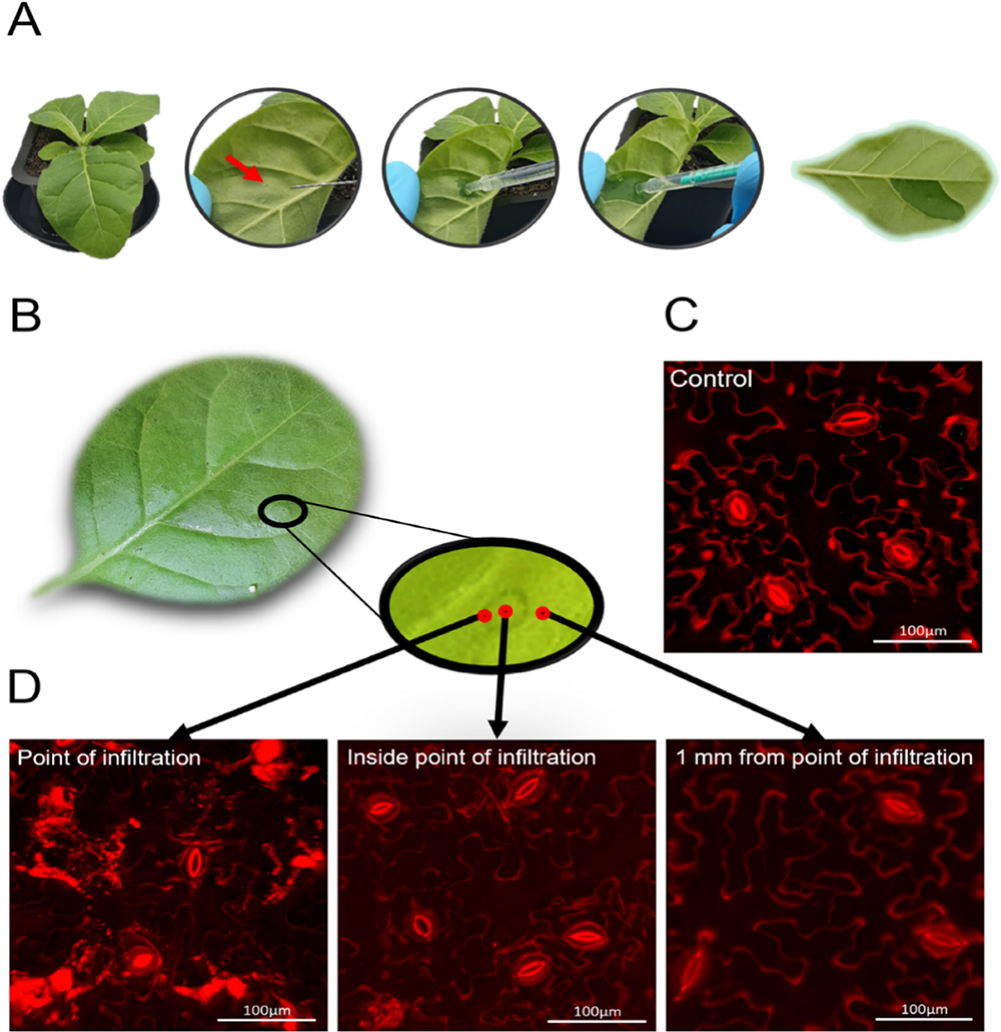
Cell viability and structural integrity of *Nicotiana tabacum*(tobacco) plants after infiltration with a MES buffer. (A) Syringe infiltration process of a tobacco leaf with a MES buffer solution. A 6-week-old tobacco plant is first placed under high luminosity (250 μmol m^–2^ s^–1^) to open the stomata. Then, a small incision is made on the abaxial side of the leaf using the tip of a syringe. Using a needleless syringe, the MES buffer solution is gently infiltrated into the leaf’s apoplast by applying pressure and maintaining the syringe at the incision site. Infiltration is stopped when the compartment of the leaf delineated by the veins is fully infiltrated (∼100 μL of buffer solution). (B) Photograph of a tobacco leaf infiltrated with a MES buffer solution with an inset showing the abaxial part of the leaf where the infiltration was performed using a needleless syringe. (C) Confocal microscopy imaging of a control tobacco leaf after staining with propidium iodide, a cationic dye that does not cross intact membranes but binds to cell walls, forming an outline of living cells. Scale bar: 100 μm. (D) Confocal microscopy imaging of a tobacco leaf infiltrated with an MES buffer. From left to right: the point of infiltration where the needleless syringe was in contact with the leaf, inside the circle formed by the pressure of the needleless syringe on the leaf tissue, and 1 mm away from the point of infiltration. Scale bars: 100 μm.

The syringe infiltration process is outlined step-by-step in Figure 1A. First, a well-watered, 6-week-old *Nicotiana tabacum* (tobacco) plant was placed under a relatively high luminosity of 250 μmol m^–2^ s^–1^ to ensure the opening of stomata. A small incision was then made on the abaxial side of the leaf, and approximately 100 μL of a 10 mM MES, 10 mM MgCl_2_ buffer solution (pH 5.6) was gently infiltrated into the apoplast of the leaves using a needleless syringe. Infiltration ceased when the leaf compartment delineated by the veins was fully infiltrated. Initially, we assessed cell viability and integrity using propidium iodide (PI) staining, a cationic dye that selectively binds to plant cell walls and does not penetrate intact cell membranes, creating a distinct outline of viable living cells.^30,31^ PI staining revealed no effect on cell viability and integrity with the exception of the cells localized at the site of the syringe infiltration, where the needleless syringe made contact with the leaf (Figure 1B–D). In this area, the tissue was disrupted, and the cells were clearly damaged. However, the rest of the infiltrated area remained undamaged, and the infiltrated plants continued to develop over the course of 4 weeks. (Figure S1).

**Figure 2:**
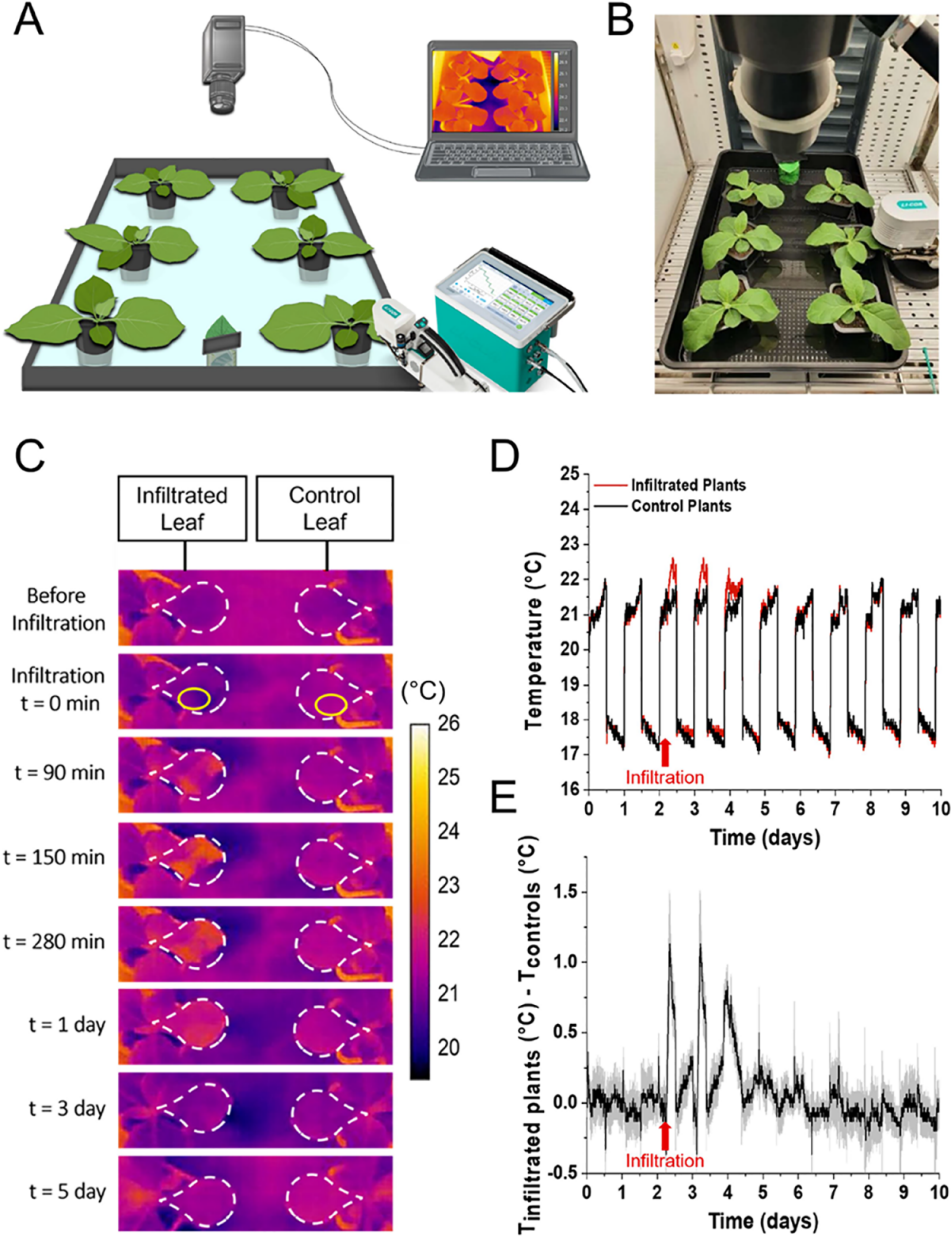
Thermal imaging of*Nicotiana tabacum* plants after infiltration with a MES buffer. (A) Schematic and (B) photograph of the experimental setup for three infiltrated and three control plants. The six plants are monitored using an infrared camera for thermal imaging, while one control plant is monitored using a portable photosynthesis system (Li-Cor LI-6800). (C) Thermal images of a tobacco leaf before and after infiltration with a MES buffer solution. The leaf on the left is infiltrated while the leaf on the right is a control leaf. The white dashed lines delineate the leaves under study, while the yellow circles represent the region of interest selected for the temperature analysis. (D) Temperature evolution of tobacco leaves after infiltration with a MES buffer solution. The red curve represents the average temperature from three infiltrated plants, and the black curve represents the average temperature from three control plants. (E) Average difference in temperature evolution between the infiltrated plants (*n* = 3) and the controls (*n* = 3). The gray outline represents the standard deviation. The red arrow indicates the time point of infiltration.

Having validated the cell viability postinfiltration with a typical MES buffer, we then investigated the impact of the infiltration process on transpiration. During infiltration, the apoplast is flooded with liquid, which may cause stomata closure and reduce transpiration. Infrared thermography is widely used to assess plant evapotranspiration, particularly on a large scale and in field conditions, as the temperature of leaves is affected by water evaporation through the cuticle and stomata (transpiration).^32−34^ With thermography, it is also possible to assess the relative stomatal conductance, which reflects how opened or closed the stomata are.^35^ Therefore, we monitored with thermal imaging the temperature changes of infiltrated and control leaves (Figure 2A,B)**.**

Thermal imaging was performed every 300 s, starting 2 days before infiltration and continuing up to 8 days after infiltration. Upon infiltration with the MES buffer solution, the leaves initially exhibited cooling in the area where the solution was introduced, which can be attributed to water evaporation from the infiltrated solution. Approximately 1 h after infiltration, both the infiltrated region and its surroundings (noninfiltrated) experienced a significant increase in temperature, attributed to a decrease in stomatal conductance and a sign of stress resulting from the infiltration process (Figure 2C). This temperature increase coincided with a distinct bending of the leaf, suggesting possible alterations in turgor pressure and cell water content due to the infiltration process and solvent evaporation from the intercellular space^36^ (Figure S2 for video). This phenomenon gradually subsided, and the leaf eventually returned to its normal position after 5 h, with the temperature gradually returning to baseline levels over the following days (Figure 2D,E). Over a set of 5 experiments, we observed an initial temperature increase between 0.8 and 1 °C, followed by recovery of the temperature 3 to 6 days postinfiltration (Figures S3 and S4).

While temperature changes correlate with plant transpiration, to gain further insight into the effect of infiltration, we calculated the relative stomatal conductance (*I*_g_). Previously, it has been shown that temperature can be converted to relative stomatal conductance with the use of dry and wet references.^37,38^
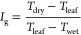
1*I*_g_, relative stomatal conductance; *T*_leaf_, Leaf temperature; *T*_dry_, Dry reference temperature; *T*_wet_, Wet reference temperature.

**Figure 3:**
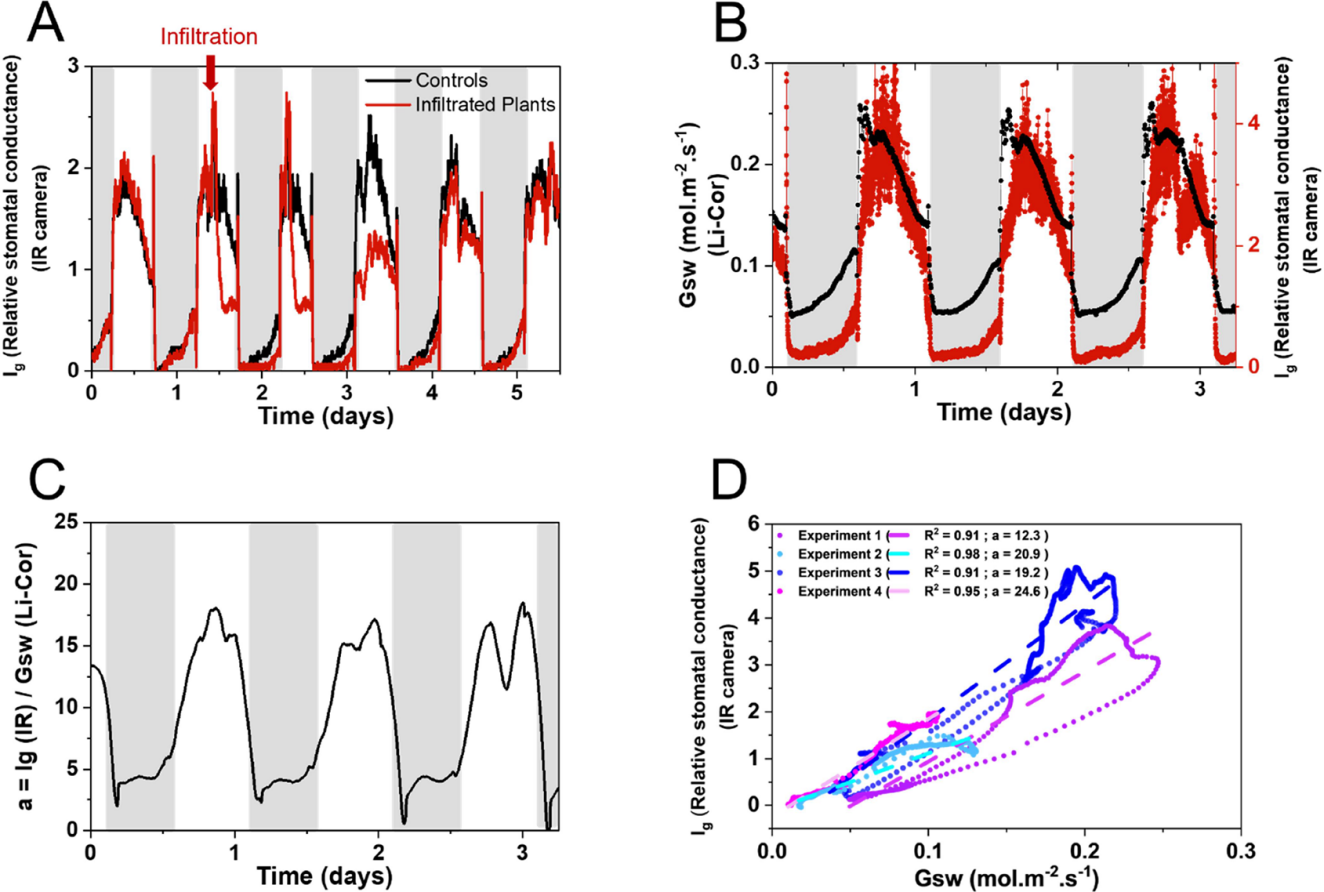
Relationship between absolute Gsw and relative *I*_g_stomatal conductance. (A) Average relative stomatal conductance *I*_g_, calculated from temperature, for control (*n* = 3) and infiltrated plants (*n* = 3). (B) Gsw of a control plant and average *I*_g_ of control plants (*n* = 3) over time. (C) Calculated ratio of *I*_g_ and Gsw over time with *prior* smoothing of the data points using the Savitsky-Golay method with a window size of 20 points. (D) Scatter plots for four different experiments of the average relative stomatal conductance *I*_g_ of control plants (*n* = 3) versus the absolute stomatal conductance Gsw, demonstrating the linear proportionality between the two parameters (smoothed data). The dotted lines represent the linear fits for each experiment, with *R*^2^ and the proportionality constant (*a*) values indicated in the legend.

*I*_g_ has been demonstrated to be directly proportional to leaf stomatal conductance in the case of isolateral leaves with equal stomatal conductance on each leaf side, such as tobacco leaves, and with the use of proper references. We observed that the calculated *I*_g_ for the infiltrated plants increases immediately following the infiltration of the MES buffer, which can be attributed to water evaporation from the infiltrated solution, but then rapidly decreases compared to noninfiltrated control plants (Figure 3A), signifying stomatal closure. However, obtaining precise absolute stomatal conductance values directly from *I*_g_ is not straightforward, as it requires additional input values such as leaf boundary layer resistance to water vapor, leaf dimensions, wind speed, and other environmental factors.^39^ Instead, we directly measured the absolute stomatal conductance (Gsw) with a portable photosynthesis fluorometer/porometer (Li-Cor LI-6800) and compared it with the relative stomatal conductance extracted through thermal imaging (*I*_g_) in control plants. To ensure that all environmental parameters were the same, we performed the two measurements simultaneously on different plants placed in the same growth environment. The direct comparison of *I*_g_ and Gsw revealed similar trends, and both curves exhibited comparable patterns, indicating that thermal imaging can provide valuable insights into stomatal conductance changes, especially when screening a large set of plants (Figure 3B).

To investigate if Gsw is directly proportional to *I*_g_ in our experiment, we calculated the ratio (*I*_g_/Gsw) for every time point. We observed that the *I*_g_/Gsw ratio changed over time, indicating that the relationship between the two parameters is time-dependent. Qualitatively, we observed that the time dependence of the *I*_g_/Gsw ratio follows a strong daily pattern in some cases (Figures 3C and S5). Furthermore, we plotted the values of the absolute stomatal conductance Gsw as a function of the calculated relative stomatal conductance *I*_g_. We observed that the curve could be fitted with a linear fit (*R*^2^ values between 0.91 and 0.98); however, the proportionality constant (*a*) was not the same for all experiments, with values lying between 12.3 and 24.6. This suggests that we cannot extract a universal proportionality constant, and therefore, we can only treat the *I*_g_ data qualitatively (Figure 3D **and full sets of experiments untreated and smoothed in**Figures S5 and S6).

After observing that the infiltration affects the stomatal conductance for a few days before recovery, we investigated its impact on the photochemical parameters of the plants. Photosynthetic parameters serve as critical indicators of plant health and performance, offering insights into the efficiency of photosynthesis and overall photosynthetic capacity.^40−42^ Specifically, we focused on the maximum quantum efficiency (*F*_v_/*F*_m_) and the quantum yield of photosystem II (ΦPSII), widely used and described as important parameters to measure the stress response of plants.^43−45^

**Figure 4:**
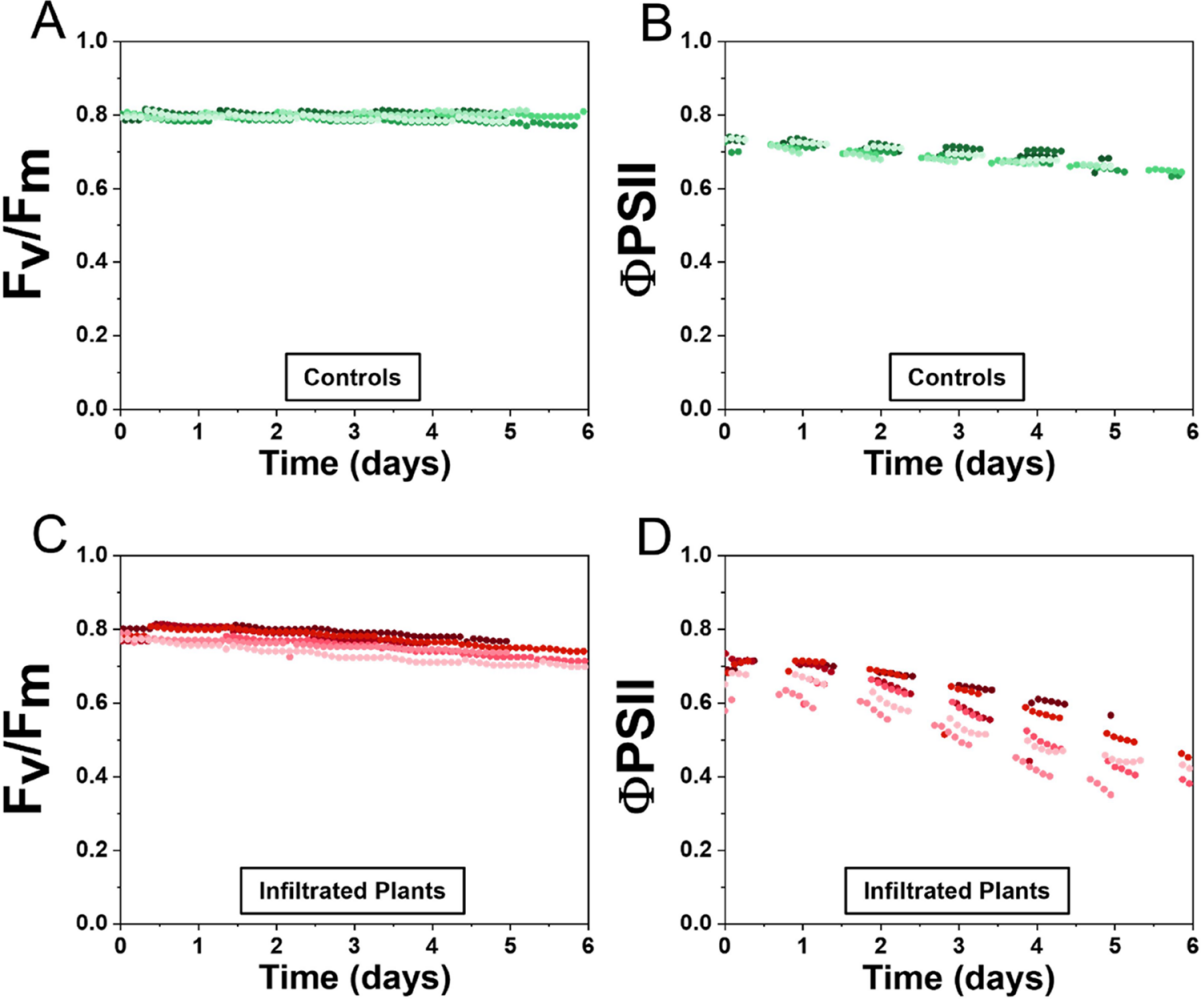
Temporal Evolution of photochemical parameters after infiltration of *Nicotiana tabacum* with MES buffer. The maximum quantum efficiency *F*_v_/*F*_m_ and the quantum yield of PSII (Φ_PSII_) were determined for controls and plants infiltrated with an MES buffer solution. Each line represents a different plant. *F*_v_/*F*_m_ (A) and Φ_PSII_ (B) evolution over 5 or 6 days for control plants without infiltration. *F*_v_/*F*_m_ (C) and Φ_PSII_ (D) evolution over 5 or 6 days for tobacco leaves infiltrated with a MES buffer solution.

To assess the photochemical changes induced by infiltration, we utilized a portable photosynthesis fluorometer/porometer system (Li-Cor LI-6800) to directly measure *F*_v_/*F*_m_ and ΦPSII. The measurements were taken every 2 h over a 5-day period on both control plants without infiltration and tobacco leaves infiltrated with the MES buffer solution (Figure 4). In control plants, we observed no significant fluctuation in *F*_v_/*F*_m_ values over the 5-day period, with a calculated variation of 0.03 ± 0.98%. In contrast, tobacco leaves infiltrated with the MES buffer solution displayed a significant change in *F*_v_/*F*_m_ values, showing a decrease of 6.06 ± 1.47% over the 5-day period. Similarly, ΦPSII values showed a relatively stable pattern in control plants, with an overall decrease of 8.59 ± 2.05% over time, likely due to stress induced by the fluorometer chamber requiring continuous contact with the leaf area. In contrast, infiltrated tobacco leaves exhibited a much larger variation in ΦPSII, demonstrating a substantial decrease of 34.04 ± 8.01%. The notable decrease in *F*_v_/*F*_m_ and ΦPSII values in the infiltrated leaves suggests a significant impact on the photochemical efficiency of the photosystem II, which could be attributed to the infiltration of the MES buffer solution. Indeed, the infiltration potentially alters the physiological state of plant cells through changes in cell turgor pressure, and as previously discussed, the reduction in stomatal conductance leads to a decreased uptake of carbon dioxide by the plants, resulting in decreased photosynthetic activity.

Additional measurements of nonphotochemical quenching (NPQ and qN), and photochemical quenching (qP) further support our findings. Control plants exhibited consistently high qP values, which decreased slightly due to the measurement process, while infiltrated plants showed a more pronounced decline. qN and NPQ values for infiltrated plants also displayed higher variability in contrast to control plants for which it remained stable (Figure S7). Moreover, estimated electron transport rates (ETR) and the ratio of open reaction centers (qL) revealed significant declines in infiltrated plants compared to controls (Figure S8). These photochemical measurements indicate that the infiltrated plants are experiencing greater stress, leading to increased nonphotochemical quenching as a protective response to excess energy, ultimately impairing their photosynthetic efficiency. A critical observation is the lack of recovery in photosynthetic activity over the 5-day period for the infiltrated leaves, despite their visually green appearance.

To summarize, in this study, we investigated the effect of syringe infiltration on *Nicotiana tabacum* (tobacco) plants using a typical 2-(N-morpholino)ethanesulfonic (MES) buffer solution without any active substances. Our results shed light on the effect of the infiltration process per se, offering valuable insights into physiological changes that occur when introducing foreign materials into plant tissues with infiltration. The cell viability and morphology analyses using propidium iodide (PI) staining revealed that the infiltration process caused localized cell damage at the site of infiltration, primarily due to compression caused by the needleless syringe. However, the overall cell viability and tissue integrity remained largely unaffected. Thermal imaging proved to be a powerful noninvasive tool for monitoring the impact of infiltration on plant physiology, especially when monitoring simultaneously a large set of plants. Our experiments demonstrated that the infiltration process temporarily altered the stomatal conductance and potentially induced changes in turgor pressure and cell water content, as we observed leaf bending. These changes in stomatal conductance were corroborated by measurements of the absolute stomatal conductance using a porometer. The use of both thermal imaging and a porometer revealed that the ratio between relative stomatal conductance and absolute values is time-dependent. However, when considering a large set of data, a proportionality constant can be extracted with acceptable fitting parameters. The proportionality constant, though, varied from experiment to experiment, preventing the establishment of a universal relationship between the two values. Nevertheless, the utilization of noninvasive techniques such as thermal imaging offers the potential for high-throughput screening and long-term monitoring of plant responses, providing valuable insights into stomatal behavior under various conditions. Although variations in wet and dry reference materials might influence the magnitude of the results, these changes are less likely to impact the observed trends, particularly in controlled environments. However, some studies suggest the need for further standardization of wet and dry reference materials, especially for large-scale plant screenings in field conditions.^46^

Finally, by monitoring the photochemical parameters of infiltrated tobacco leaves, we observed a significant decrease in maximum quantum efficiency (*F*_v_/*F*_m_) and quantum yield of photosystem II (ΦPSII) values over the 5-day period. This decrease can be linked to the reduced stomatal conductance, leading to a decline in carbon dioxide uptake and subsequent photosynthetic activity.^47−50^ Notably, in the infiltrated leaves, the photosynthetic activity did not recover over the 5-day observation period, suggesting sustained changes in photosystem efficiency. This sustained decrease in photosynthetic activity warrants further investigation to understand the underlying mechanisms and potential implications for plant biotechnology applications. In this study, we utilized 5- to 6-week-old tobacco leaves of similar size, shape and position for infiltration experiments to reduce variability in the measurements, and as this age is optimal for size, health, and ease of manipulation. Although this is a common age range used in similar studies, further investigations using older leaves could provide additional insights into how leaf age influences infiltration effects. This could be particularly valuable for optimizing the methodology in future research. Additionally, future experiments involving longer observation periods would be beneficial to determine if the infiltrated leaves maintain their chlorophyll content, as the leaves appear visually green despite the altered photosynthetic parameters. This investigation would provide essential insights into whether the photosynthetic alterations are solely related to changes in chlorophyll content or if other factors contribute to the observed changes in photochemical parameters.

This work highlights the importance of considering the impact of the infiltration process *per se* when studying the effects of infiltrated substances on plant biology. While this study relied on simple and noninvasive methods for studying the impact of syringe infiltration using a typical MES buffer, future research can explore additional molecular and biochemical analyses, such as gene expression profiling, metabolomic profiling, and enzymatic activity assays, to gain a comprehensive understanding of the effects of infiltration on cellular processes and metabolic pathways. Furthermore, investigating the response of different plant species and varying infiltration mediums will broaden our understanding of the generalizability of these findings. Nonetheless, in light of the results from this study, several potential optimizations for the infiltration protocol can be considered for future research. While significant advancements have been made in optimizing buffers for transient gene expression, adjustments to the salt composition and pH of the MES buffer, or the inclusion of specific active substances, could improve both the osmotic balance and the physiological response of the plants. Further optimization could involve studying different buffer formulations for enhancing infiltration efficiency and reducing any unintended side effects on leaf structure and function. Additionally, environmental factors such as light intensity, temperature, and humidity could be further explored for their role in enhancing stomatal opening prior to infiltration. Infiltration technique optimizations, such as using regulated pressure devices for more consistent delivery or modifying syringe designs to reduce leaf damage, also hold promise for improving efficiency and uniformity across samples. Additionally, exploring passive infiltration methods through osmotic gradients, hydrogel-based infiltration or other carriers may also provide less invasive options.^51^ Extending these techniques to other plant species could help determine the broader applicability of these findings and further refine the protocol across different contexts. Ultimately, our findings contribute to the development of refined protocols and advancing infiltration techniques for a wide range of plant-related studies, providing a valuable approach for monitoring physiological parameters postinfiltration in large-scale experiments. By highlighting the significant physiological impacts of syringe infiltration, we encourage to carefully consider these effects when designing experiments and aim to inform about the importance of optimizing these protocols. This awareness can lead to improved data reliability and enhance the quality of physiological assessments, particularly in fields like nanobionics where precise physiological measurements are essential.

## Materials and Methods

### Plant Material and Growth Conditions

*Nicotiana tabacum* SR1 (tobacco) seeds were planted in soil and grown in a growth chamber (Percival, CLF PlantClimatics GmBH, Wertingen, Germany) under controlled conditions and a day/night cycle of 12 h. The growth chamber sustained a relative humidity of 60%, a photon flux density of 110 μmol m^–2^ s^–1^, a temperature of 24 °C during the day and 18 °C during the night, and a CO_2_ concentration of 400–420 ppm. The plants were cultivated for at least 5 weeks before being employed in all subsequent experimental procedures and measurements. For the infrared camera experiments, plants of the same age and similar size and shape were selected to reduce variability in the measurements.

### Infiltration of *Nicotiana tabacum* (Tobacco)

Before infiltration, the tobacco plants were subjected to high-intensity light at approximately 500 μmol m^–2^ s^–1^ for 1 h to ensure the opening of stomata. Then, a small hole was made on the abaxial side of the tobacco leaf using a sterile 0.8 × 50 mm HENKE-JECT syringe needle, with one hole per leaf to be infiltrated. Notably, the hole was made to penetrate only the epidermal layer, not completely through the leaf, to facilitate the subsequent infiltration process into the plant tissue. The infiltration was carried out by delivering 100 μL of a 10 mM MES, 10 mM MgCl_2_, pH 5.6, buffer solution through the hole using a needleless syringe. To maintain precise control over the applied pressure and avoid damage to the plant tissue, the researcher placed a finger on the adaxial side of the leaf to stabilize the syringe on the abaxial side. The buffer solution was then gently pushed into the leaf by pushing the syringe plunger. For photosynthetic measurements, the infiltrated plants were allowed to stand for 24 to 48 h postinfiltration under normal growth conditions, enabling any residual solvent to evaporate. For thermal imaging experiments and staining, the infiltrated plants were allowed to stand in normal growth conditions and were monitored for up to 2 weeks. This extended monitoring period provided insights into the long-term effects of infiltration on the plant’s physiological responses.

*Propidium Iodide staining* was carried out by first excising the leaf of interest with a clean razor blade. The leaf was then immersed in a 20 μmol aqueous solution of Propidium Iodide (Sigma-Aldrich, St. Louis, MO, USA) contained in a 50 mL conical tube (Sarstedt, Nümbrecht, Germany) for a duration of 20 min. After staining, the leaf was gently taken out from the solution, and any excess staining was eliminated by washing the leaf with DI water. The stained leaves were then immediately mounted for microscopy for further analysis.

### Confocal Microscopy Imaging

Confocal imaging was performed with an inverted Zeiss LSM 980 (Carl Zeiss AG, Oberkochen, Germany) confocal microscope equipped with an Airyscan2 detection unit. The objective Plan-Apochromat 63×/1.4 Oil DIC M27 (FWD = 0.19 mm, Carl Zeiss AG, Oberkochen, Germany) was used, with Immersol 518 F immersion media (*n* = 1.518, Carl Zeiss AG, Oberkochen, Germany), and all images were processed with the Zen Blue software 3.4. The images were captured in 8 bits with a bidirectional acquisition, an average of 4 images, an image size of 1984 × 1984 pixels (pixel size = 0.171 μm), and a zoom of 1. The Propidium Iodide was excited with a 561 nm laser at an intensity of 6.5%, with a pinhole of 7.8 AU, a detector gain at 630 V, a pixel time of 0.26 μs, and detected using the RedTex filter (emission wavelength 614 nm).

*Thermal images* were obtained through a ThermoVision A320G camera (FLIR Systems) with a wide-angle objective (lens *f* = 4 mm), a spectral range of 7.5–13.0 μm, and a resolution of 320 × 240 pixels. The leaf temperature evolution is monitored every 300 s starting 2 days before infiltration until up to 8 days after.

In order to convert the temperature into relative stomatal conductance, we used a dry and wet reference. The dry reference was made of a rectangular piece of metal (10 cm × 6 cm) painted with commercially available matte black paint with high absorbance to simulate a nontranspiring leaf. The wet reference was made with a piece of green cotton fabric shaped like a round leaf (∼6 cm of diameter) using a metal wire. The wet reference was constantly dipped in a beaker filled with DI water to mimic a transpiring leaf with high surface conductance.

*Stomatal and photosynthesis measurements* were performed using a portable photosynthesis fluorometer/porometer (Li-Cor LI-6800, Li-COR Inc., Lincoln, NE, USA). The chamber of the system (6 cm^2^) was set with the same humidity (60%), light intensity (110 μmol m^–2^ s^–1^), light cycle (12 h/12 h day/night), and temperature (24 °C/18 °C day/night) as in the growth chamber, with a CO_2_ concentration of 410 μmol mol^–1^. Gas exchange (stomatal) and photosynthetic measurements were not performed simultaneously for infiltrated leaves as the infiltration and evaporation of the infiltration medium seemed to impact the accuracy of the gas exchange system. Fluorescence measurements for the evaluation of the maximum quantum efficiency (*F*_v_/*F*_m_) and the quantum yield of photosystem II (ΦPSII) were conducted 1 day after infiltration for infiltrated plants and, for all plants, every 2 h using a pulsed light of 10,000 μmol m^–2^ s^–1^ to measure the minimal fluorescence in the dark (measurements performed at night) and maximal fluorescence upon saturating light pulse.

The variations for the photosynthetic parameters in Figure 4 were obtained by calculating the variation of the *F*_v_/*F*_m_ and ΦPSII values between day 1 and day 5 for each experiment following [(*X*_1_ – *X*_2_)/*X*_1_] × 100, and averaging all the variations.

## Supporting Information Available

Confocal imaging for long-term monitoring of tobacco postinfiltration; thermal imaging video showing temperature variation postinfiltration and leaf bending; unaveraged temperature evolution of a full set of plants postinfiltration for one experiment; comparison of temperature evolution between infiltrated and control plants for five different experiments; graphs illustrating the relation between absolute Gsw and relative *I*_g_ stomatal conductance, untreated and smoothed, with calculated ratio of *I*_g_ and Gsw over time; and scatter plots of the absolute stomatal conductance Gsw versus the average relative stomatal conductance *I*_g_ for each experiment (PDF)

## Supplementary Material

as4c00170_si_001.pdf

as4c00170_si_002.avi
