# Supplementary material for: Investigating the Effect of Syringe Infiltration on Nicotiana tabacum (Tobacco)
Source: ACS Agric Sci Technol. 2024 Dec 21;5(1):28–35. doi: 10.1021/acsagscitech.4c00170 (PMC11752493; doi:10.1021/acsagscitech.4c00170)
Supplement: Supplementary file 1 — as4c00170_si_001.pdf [file as4c00170_si_001.pdf]

Supporting Information

Investigating the effect of syringe infiltration on *Nicotiana tabacum* (tobacco)

Cyril Routier[1], *Carmen Hermida-Carrera*[2], *Eleni Stavrinidou*[1,2]*

[1] Laboratory of Organic Electronics, Department of Science and Technology, Linköping University, SE-60174, Norrköping, Sweden.

[2] Umeå Plant Science Centre, Department of Forest Genetics and Plant Physiology, Swedish University of Agricultural Sciences, SE-90183 Umeå, Sweden.

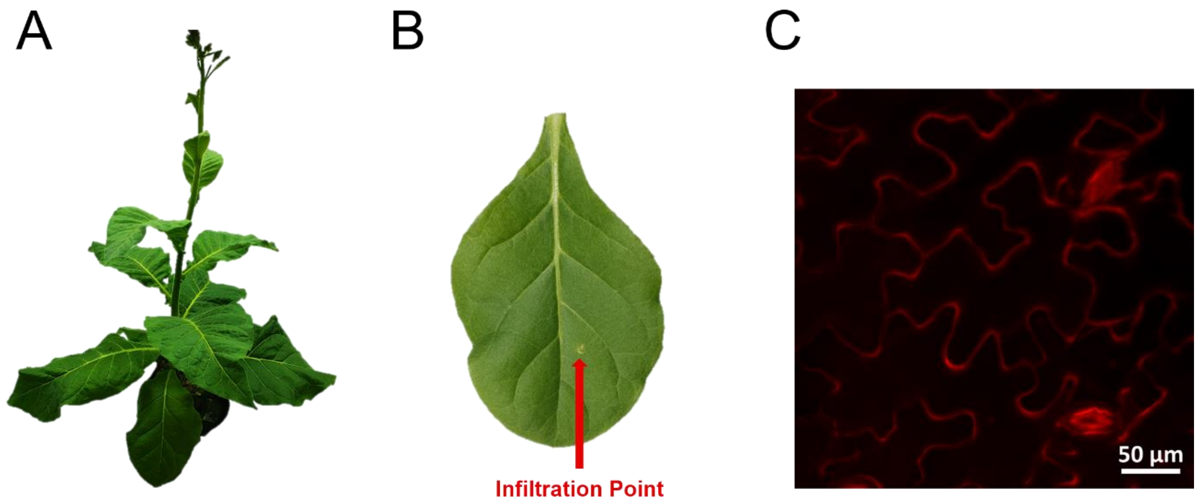

Figure S1: Long-term monitoring of the growth of a *Nicotiana tabacum* (tobacco) plant infiltrated with a typical MES buffer. (A) Picture of a plant one month after infiltration with a MES buffer, the plants grows and develop flowers normally. (B) The leaf infiltrated is still green and healthy looking 1 month after infiltration with the exception of a yellow dry spot where the syringe was in contact with the tissue and compressed the cells. (C) Confocal microscopy imaging of a leaf 1 month after infiltration with an MES buffer and after staining with propidium iodide (PI). Scale bar: 50 µm

Figure S2 (Video added as a file): Video of infiltrated and control leaves captured through an infrared camera showing the temperature evolution and bending of the leaves after infiltration.

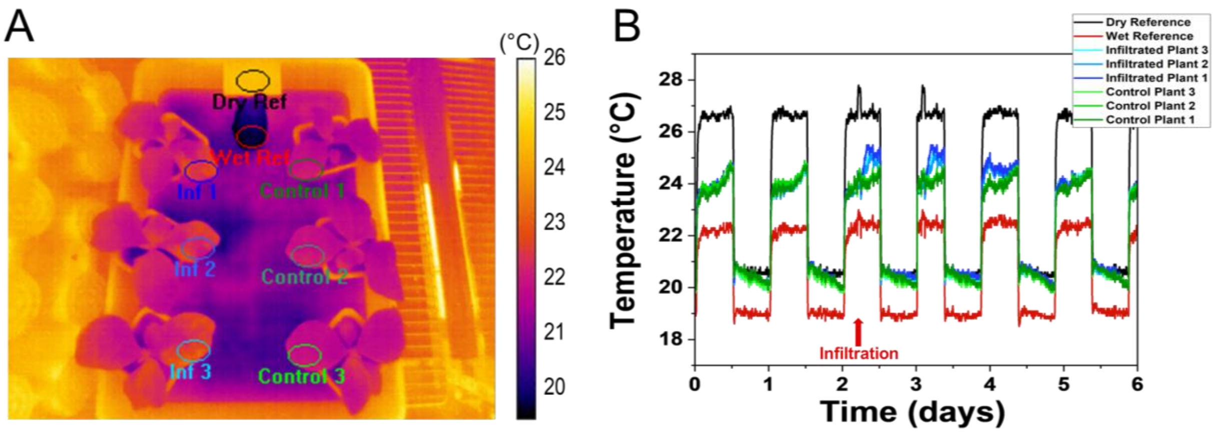

Figure S3: (A) Thermal image of a classic experimental setup including 3 plants infiltrated with a typical MES buffer (inf 1-3), 3 controls (control 1-3), and dry and wet references (Dry Ref and Wet ref). The DOIs are also presented in the image, similar in pixel density and always centered around the point of infiltration. Although for long experiments, as the leaves are moving and growing, it may be required to adjust the position of the DOIs or even part the videos in several shorter ones. (B) Temperature evolution extracted from an infrared camera for 3 plants infiltrated with a typical MES buffer, 3 controls, and wet and dry references. The relative stomatal conductance can then be calculated for each plant monitored by following the equation (1) in the main text : $I_g=(T_{dry}-T_{leaf})/(T_{leaf}-T_{wet})$.

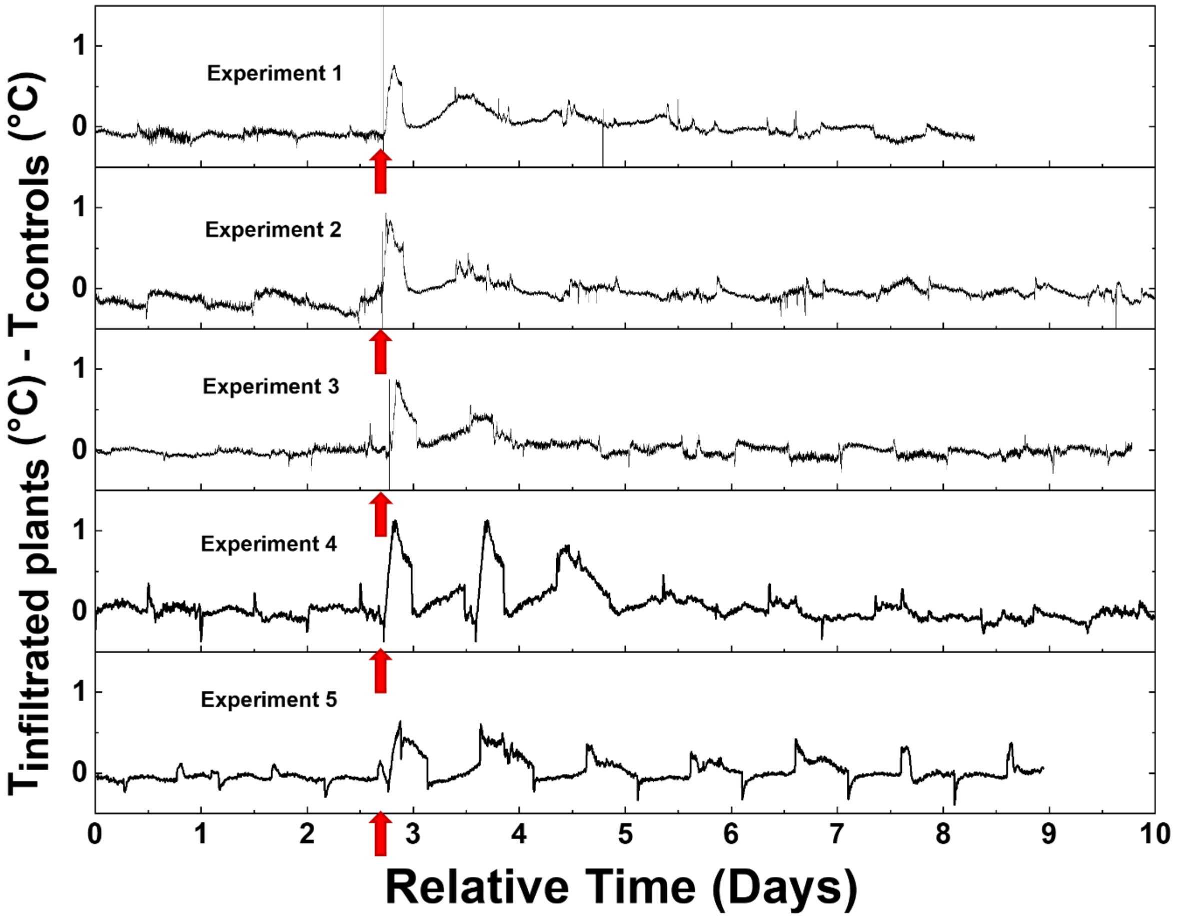

Figure S4: Difference in temperature evolution between plants infiltrated with a typical MES buffer and controls plants. Five sets of experiments are represented here. Each experiment includes 3 controls and 3 infiltrated plants. The red arrow indicates the time point of infiltration.

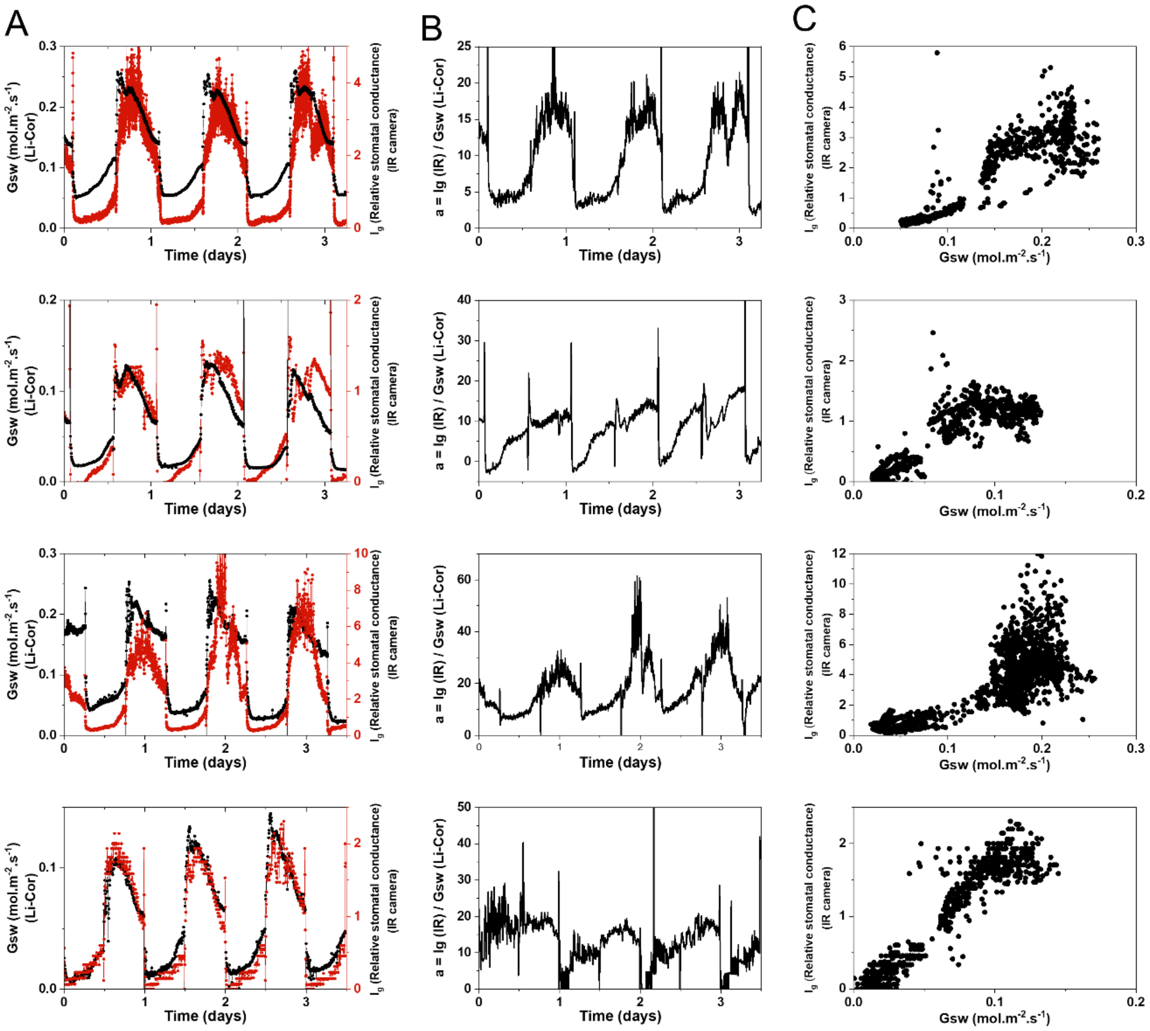

Figure S5: Full set of experiments for the relation between absolute Gsw and relative I_g stomatal conductance. Each row represents an independent experiment**.** (A) Gsw of a control plant and average I_g of control plants (n=3) over time. (B) Calculated I_g/Gsw ratio over time. (C) Scatter plots of the average relative stomatal conductance I_g of control plants (n=3) versus absolute stomatal conductance Gsw of a control plant for the entire length of the measurement (non-smoothed data).

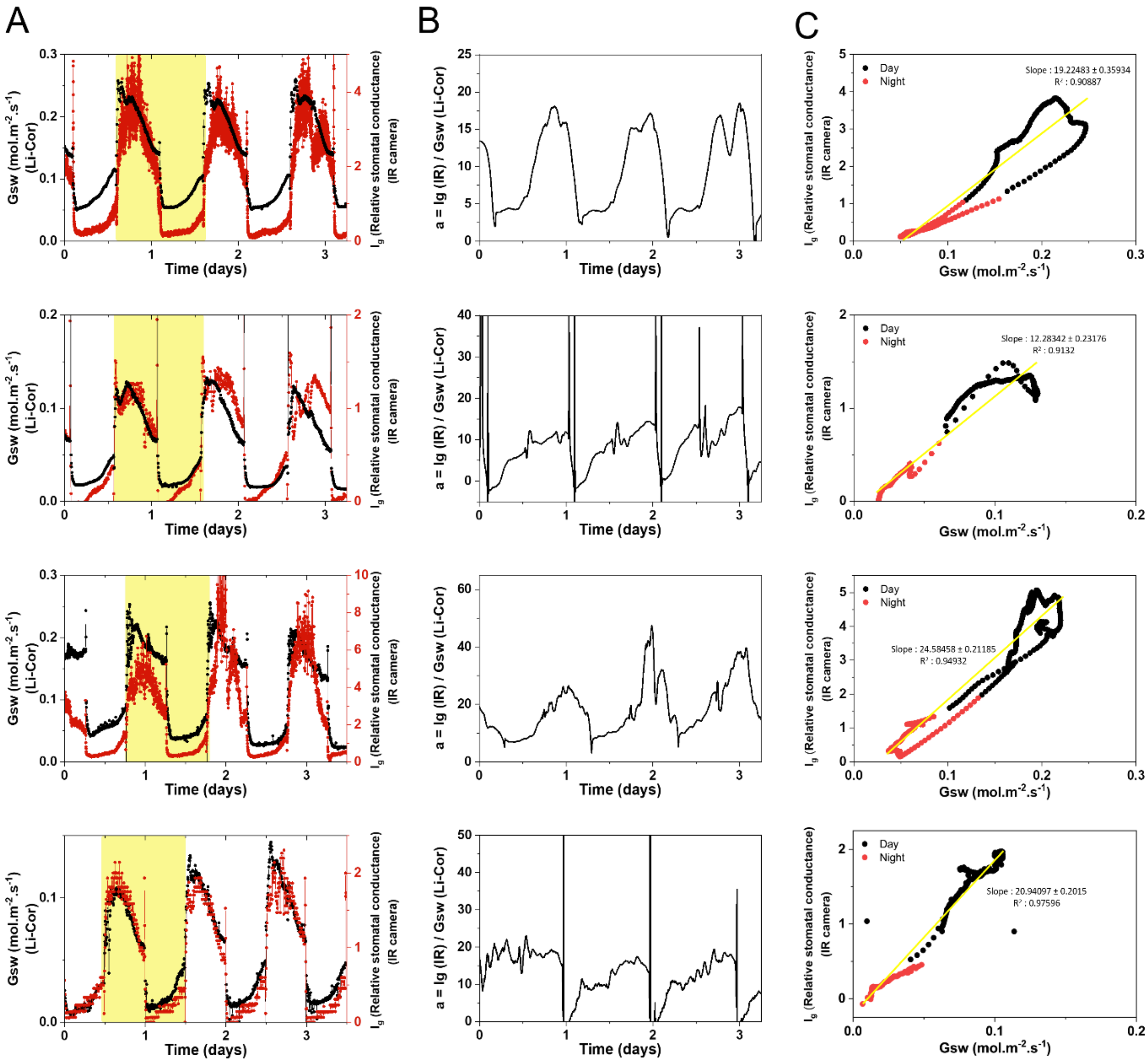

Figure S6: Full set of experiments for the relation between absolute Gsw and relative I_g stomatal conductance with smoothed datasets. Each row represents an independent experiment (A) Gsw of a control plant and average Ig of control plants (n=3) over time. The yellow band indicates the day used for the plots in column C. (B) Calculated I_g/Gsw ratio over time with *prior* smoothing of the data points using the Savitsky-Golay method with a window of 20 points. (C) Scatter plots of the average relative stomatal conductance I_g of control plants (n=3) versus the absolute stomatal conductance Gsw of a control plant (smoothed data corresponding to day 1, marked in yellow in column A). The linear fit is represented in yellow with the R^2 and slope indicated in the graphs, demonstrating the linear proportionality between the two parameters.

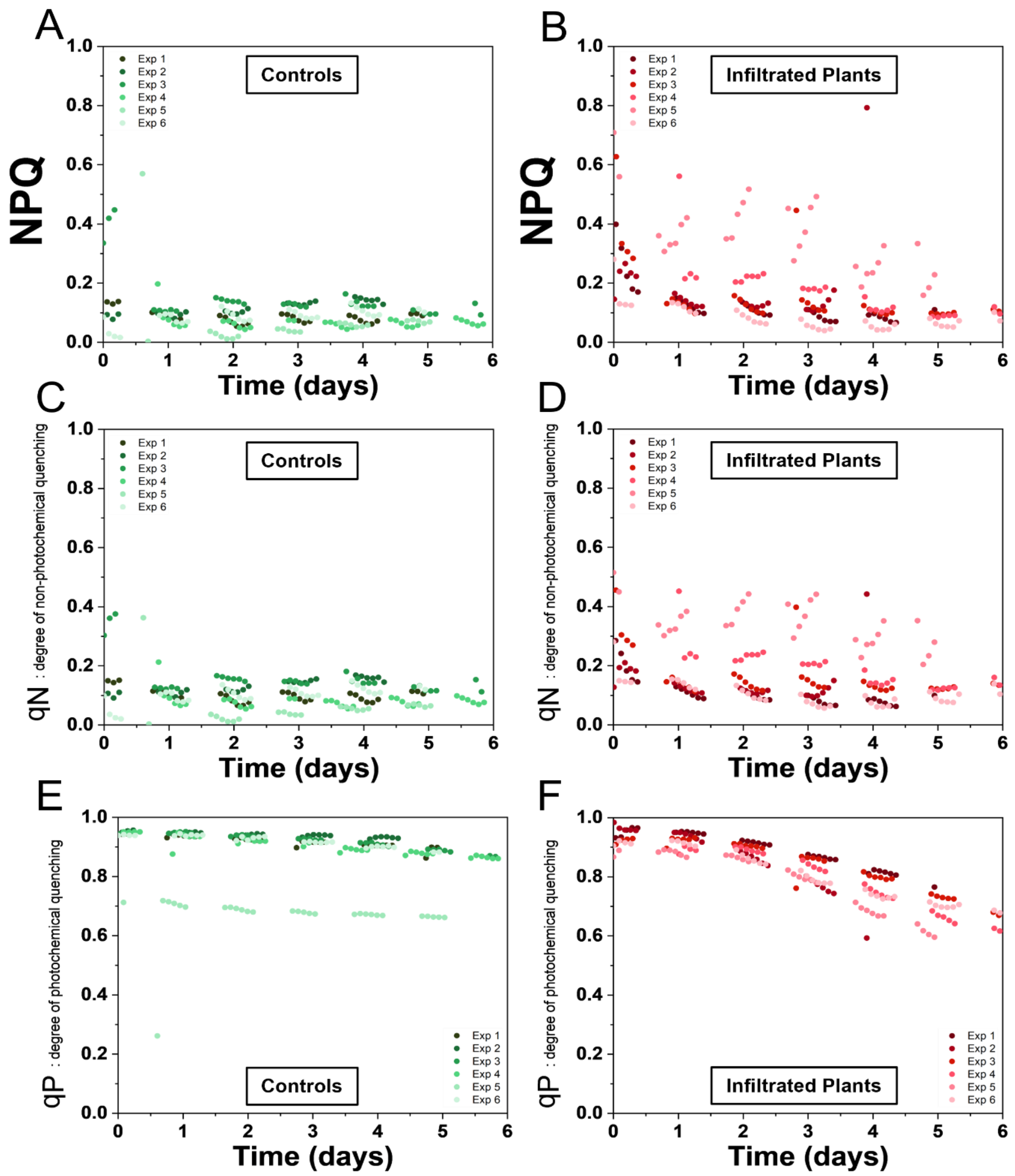

Figure S7: Temporal Evolution of non-photochemical and photochemical quenching parameters after infiltration of *Nicotiana tabacum* with MES buffer. (A) and (B); Non-photochemical chlorophyll fluorescence quenching (NPQ) for controls and plants infiltrated with an MES buffer solution. Each line represents a different plant. The NPQ is not bound between 0 and 1 and reflects the energy dissipation as heat. (C) and (D); Non-Photochemical Quenching Coefficient (qN) for controls and plants infiltrated with an MES buffer solution. Each line represents a different plant. The qN reflects non-photochemical energy dissipation and is bound between 0 and 1. (E) and (F); Photochemical Quenching Coefficient (qP) for

controls and plants infiltrated with an MES buffer solution. Each line represents a different plant. The qP reflects the fraction of open PSII reaction centers (**Figure S8C** and **S8D**). High qP values (close to 0.9) indicate that most reaction centers are open and functional, which is common under non-stressful, well-lit conditions.

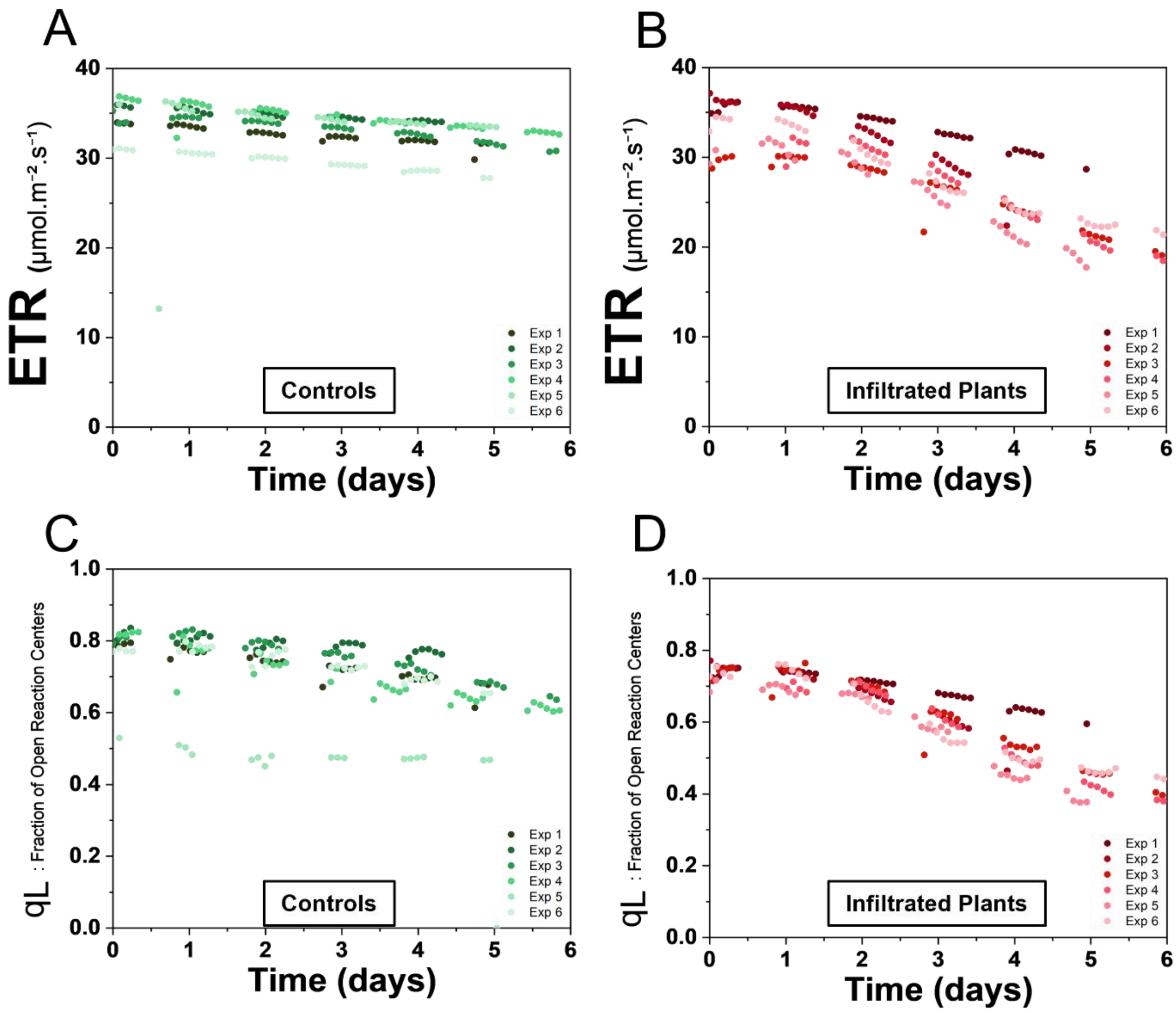

Figure S8: Temporal Evolution of the estimated rate of electron transport (ETR) fraction of open reaction centers (qL) after infiltration of *Nicotiana tabacum* with MES buffer. (A) and (B); estimate of the rate of electron transport (ETR) through photosystem II (PSII) for controls and plants infiltrated with an MES buffer solution. The ETR is directly related to the photosynthetic activity and the light reactions of photosynthesis. Each line represents a different plant. Higher ETR values generally indicate more efficient light capture and use in photosynthesis. (C) and (D); estimate of the fraction of open reaction centers (qL) for controls and plants infiltrated with an MES buffer solution. Each line represents a different plant. Estimated from the qP in **Figure S7E** and **S7F**.
